# Engineering analysis of aortic wall stress and root dilatation in the V-shape surgery for treatment of ascending aortic aneurysms

**DOI:** 10.1093/icvts/ivac004

**Published:** 2022-02-03

**Authors:** Hai Dong, Minliang Liu, Tongran Qin, Liang Liang, Bulat Ziganshin, Hesham Ellauzi, Mohammad Zafar, Sophie Jang, John Elefteriades, Wei Sun

**Affiliations:** 1 Tissue Mechanics Laboratory, The Wallace H. Coulter Department of Biomedical Engineering, Georgia Institute of Technology and Emory University, Atlanta, GA, USA; 2 Department of Computer Science, University of Miami, Coral Gables, FL, USA; 3 Aortic Institute at Yale New Haven Hospital, Yale University School of Medicine, New Haven, CT, USA

**Keywords:** V-shape surgery, Ascending aneurysm, Aortic root, Wall stress, Finite element analysis

## Abstract

**OBJECTIVES:**

The study objective was to evaluate the aortic wall stress and root dilatation before and after the novel V-shape surgery for the treatment of ascending aortic aneurysms and root ectasia.

**METHODS:**

Clinical cardiac computed tomography images were obtained for 14 patients [median age, 65 years (range, 33–78); 10 (71%) males] who underwent the V-shape surgery. For 10 of the 14 patients, the computed tomography images of the whole aorta pre- and post-surgery were available, and finite element simulations were performed to obtain the stress distributions of the aortic wall at pre- and post-surgery states. For 6 of the 14 patients, the computed tomography images of the aortic root were available at 2 follow-up time points post-surgery (Post 1, within 4 months after surgery and Post 2, about 20–52 months from Post 1). We analysed the root dilatation post-surgery using change of the effective diameter of the root at the two time points and investigated the relationship between root wall stress and root dilatation.

**RESULTS:**

The mean and peak max-principal stresses of the aortic root exhibit a significant reduction, P=0.002 between pre- and post-surgery for both root mean stress (median among the 10 patients presurgery, 285.46 kPa; post-surgery, 199.46 kPa) and root peak stress (median presurgery, 466.66 kPa; post-surgery, 342.40 kPa). The mean and peak max-principal stresses of the ascending aorta also decrease significantly from pre- to post-surgery, with P=0.004 for the mean value (median presurgery, 296.48 kPa; post-surgery, 183.87 kPa), and P=0.002 for the peak value (median presurgery, 449.73 kPa; post-surgery, 282.89 kPa), respectively. The aortic root diameter after the surgery has an average dilatation of 5.01% in total and 2.15%/year. Larger root stress results in larger root dilatation.

**CONCLUSIONS:**

This study marks the first biomechanical analysis of the novel V-shape surgery. The study has demonstrated significant reduction in wall stress of the aortic root repaired by the surgery. The root was able to dilate mildly post-surgery. Wall stress could be a critical factor for the dilatation since larger root stress results in larger root dilatation. The dilated aortic root within 4 years after surgery is still much smaller than that of presurgery.

**Figure:**
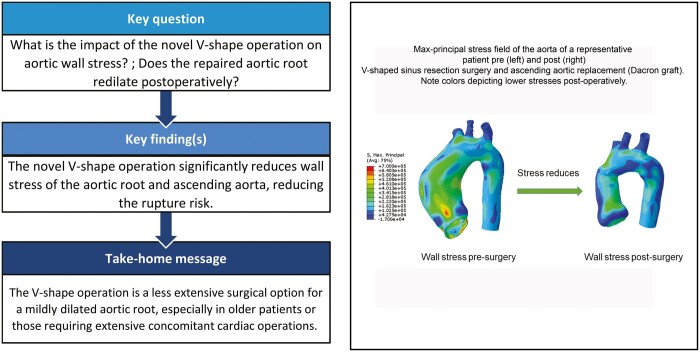

## INTRODUCTION

Ascending aortic aneurysms often include dilatation of the sinotubular junction (STJ) and extend proximally into the root portion of the aorta. In the root zone, it is the non-coronary sinus that dilates earliest and most severely (thought due to lack of structural support by any coronary ostium) [1, 2], which often leads to aortic insufficiency [3, 4]. Root pathology can be eradicated by traditional surgeries such as full aortic root replacement or valve-sparing aortic root replacement. However, this magnitude of surgical procedure may represent excessive surgical intervention for infirm or elderly patients. We recently [5] reported a simpler V-shape resection technique of the non-coronary sinus, which could reduce the diameter and cross-sectional area of the aortic root aneurysm.

Biomechanical factors such as aortic wall stress play a fundamental role in the natural history of aneurysms [6–8]. Based on engineering principles of material failure [9–16], aneurysm rupture and dissection occur when aortic wall stress exceeds the yield and failure strength criteria of the vessel wall. Moreover, the aortic wall stress is also highly related to the dilatation of aortic aneurysms [17, 18]. Wall stress analysis can clarify the bioengineering impact of V-shaped root resection.

The well-known Laplace’s law is a simple method to calculate the aortic wall stress, which provides a rough estimation. The finite element (FE) method, based on patient-specific geometry, can be applied to obtain more accurate stress values [19–21]. The aim of this study was to assess the morphologic and biomechanical improvements accomplished by the novel V-shape surgery based on the FE analysis.

## MATERIALS AND METHODS

### Ethics statement

This retrospective chart review study was approved by the Georgia Tech Institutional Review Board (#H20373, 13 October 2020) and Yale University Human Investigation Committee (#2000024367, 12 November 2018). Individual consent was waived.

### Study design

We retrospectively collected clinical cardiac computed tomography (CT) images of 14 patients (P1–P14) who underwent V-shape resection of the non-coronary sinus, together with supracoronary ascending aortic replacement, at Yale New Haven Hospital, New Haven, CT, between 2013 and 2018. Patients were selected for this surgery based on their anatomy and other findings in the operating room. Patients with severe enlargement of the aortic root and sinuses of Valsalva underwent full replacement of the aortic root by conventional techniques. Patients with only moderate enlargement of the non-coronary sinus were selected, based on surgeon’s judgement at the operating table, for the V-shape resection procedure. Among those patients, all with suitable imaging studies underwent analysis in the present report. The patients’ inclusion was consecutive per the above criteria. For 10 (P1–P10) of the total 14 patients, the CT images of the thoracic and abdominal aorta pre- and post-surgery were obtained. We used these 10 patients to investigate the impact of the V-shape surgery on the stress distribution of the aortic wall. For 6 (P1, P8, and P11–P14) of the total 14 patients, CT images of the aortic root at 2 follow-up time points post-surgery were available. We used these 6 patients to study the dilatation of the aortic root post-surgery by estimating the temporal evolution of the overall effective diameter of the aortic root.

### The V-shape surgery

**Figure 1:**
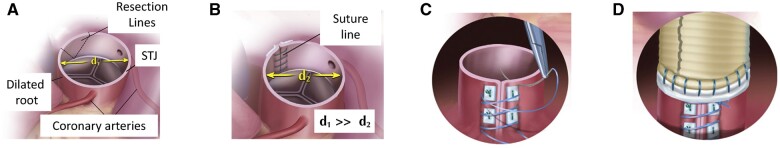
Artist’s rendition of V-shaped surgery. (**A**) Line of resection indicated. (**B**) Reapproximation after resection. (**C**) Details of first everting layer of pledgeted sutures and second running layer. (**D**) End-to-end attachment of ascending graft to remodelled aortic root. Adapted and reprinted from Ref. [5] with permission.

During the surgery, a deep V-shaped, triangular portion of the non-coronary sinus is resected, from the STJ to just above the aortic annulus (see Fig. 1). The tissue in the resected non-coronary sinus was often thin, but the two-layer closure proved secure in all patients in this small series. This simple, quick V-shape resection addresses the aortic root dilatation without the complexities of full aortic root surgery with coronary button reimplantation. In our practice, choosing the V-shape resection is the exception rather than the rule, being reserved for elderly or infirm patients or those requiring other unrelated concomitant cardiac procedures.

### Construction of patient-specific finite element model

**Figure 2:**
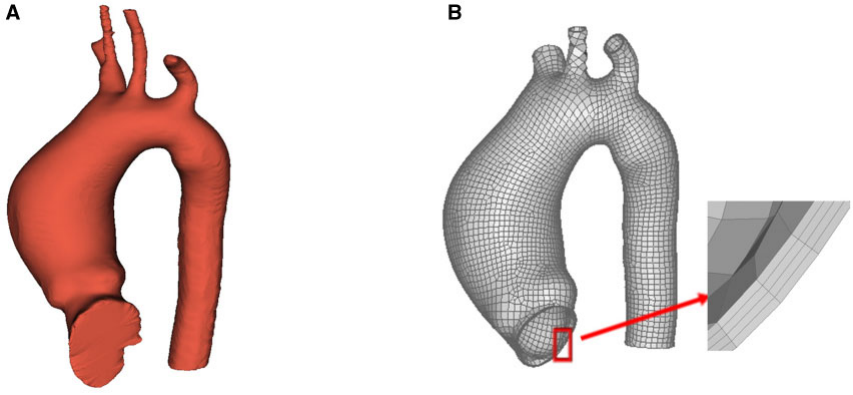
(**A**) Three-dimensional geometry of the aorta of a representative patient (P1) presurgery reconstructed in three-dimensional Slicer (www.slicer.org); (**B**) Finite element model generated in HyperMesh 2017 (Altair Engineering) based on the geometry in (**A**) with trimmed boundaries.

For each patient of P1–P10, the three-dimensional (3D) surface geometry (Fig. 2A) of the aorta was reconstructed from the CT images using 3D Slicer (www.slicer.org). The aortic root, ascending aorta and aortic arch, together with the 3 branches (the brachiocephalic artery, the left common carotid artery and the left subclavian artery), and the proximal descending aorta were obtained (Fig. 2A). The surface geometry was exported from 3D Slicer and imported into Altair HyperMesh 2017 (Altair Engineering, Troy, MI, USA) to generate the FE model for the aorta with trimmed boundaries (Fig. 2B). We first created 4-node quadrilateral shell elements with element size of about 2 mm by 2 mm based on the surface geometry. Four layers of 8-node linear brick elements (C3D8R) were created by offsetting the 4-node quadrilateral shell elements [22]. A total thickness estimate of 1.5 mm was applied to the aortic wall following Liang *et al.* [23]. Analysis of mesh size independence was performed.

### Stress calculation

The static determinacy is a property of structures whose internal tension/stress is independent of the material properties (i.e. the tension/stress can be calculated based only on the external force and geometry) [21]. Using Laplace’s law to compute the wall hoop stress of a perfect cylindrical tube is one of the most well-known examples of static determinacy [21]. Though the geometry of the aorta is much more complex, it has been shown [20, 21, 24] that the aorta under a pressure loading can also be treated as a structure with static determinacy and that the aortic wall stress is independent of material properties [25–27] and can be calculated directly from the pressure and geometry.

The stress distributions of the aortic wall under systolic pressure were obtained at pre- and post-surgery states, using the FE simulations based on the static determinacy approach [20, 21, 24]. The FE simulation of the aorta was performed by the Abaqus/Standard 2019 (SIMULIA, Providence, RI, USA). The details can be found in the Supplementary Materials.

### Dilatation of the aortic root post-surgery

**Figure 3:**
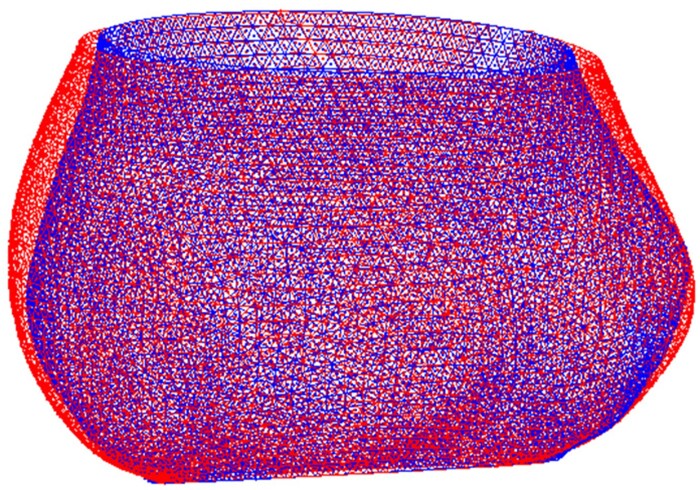
Three-dimensional geometries of the aortic root of a representative patient (P13) post-surgery at 2 follow-up time points. Post 1 (blue): about 4 months after the surgery; Post 2 (red): 20 months later from Post 1. Note minimal growth of the aortic root between time points.

For the 6 patients, i.e. P1, P8 and P11–P14, 3D geometries of the aortic root post-surgery at the 2 follow-up time points, Post 1 and Post 2, were reconstructed from the CT images using the same method described in the previous subsection of construction of patient-specific FE model. Figure 3 shows the 3D geometries of the inner surface of the aortic root (aortic annulus to STJ) of a representative patient (P13), with Post 1 (blue) at about 4 months after the surgery and Post 2 (red) at about 20 months from Post 1. The geometries in Fig. 3 indicate that the aortic root dilated minimally after the surgery. We used the effective diameter ($D_{e}$) of the aortic root at Post 1 and Post 2 for all 6 patients to estimate the overall dilatation of the aortic root post-surgery. The diameter of Post 1 was regarded as the initial diameter of the aortic root after the surgery. The details for calculation of the effective diameter and growth can be found in the Supplementary Materials. In this study, the investigated follow-up period was defined as the time interval between the surgery date and the date of Post 2 (denoting as ${\Delta T}_{02}$). The potential follow-up period was defined as the time interval between the surgery date and the study closure date (denoting as ${\Delta T}_{0c}$). The follow-up index of each patient was calculated based on ${\mathrm{FUI}={\Delta T}_{02}/\Delta T}_{0c}$. We also obtained the stress distribution of the aortic root (from around the aortic annulus to the STJ) based on the FE simulation. A linear regression was performed between the diameter growth (from Post 1 to Post 2) and the mean max-principal (MP) stress of root, with y=ax+b, where x is mean MP stress, y is the diameter growth and {a, b} are parameters to fit. The linear regression was performed using the *polyfit* function in the MATLAB 2018b (Mathworks Inc., Natick, MA, USA).

### Statistical analysis

The Wilcoxon signed-rank test was used to compare the stress from different groups (e.g. pre- and post-surgery), with the null hypothesis that the difference of the stress data pre- and post-surgery comes from a distribution with zero median. All analyses were performed with the *signrank* function in the MATLAB 2018b (Mathworks Inc., Natick, MA, USA). A *P*-value <0.05 was considered to be statistically significant.

## RESULTS

### Patient data

Patient demographics and CT findings of the 14 patients involved in this study are provided in Table 1. Most patients (11/14, 78.57%) had preoperative aortic insufficiency (AI) (mild in 4, moderate in 5 and severe in 2). The 11 patients who had preoperative AI underwent concomitant aortic valve replacement. None of the patients who underwent a valve-sparing procedure developed AI postoperatively. During the study period, a total of 21 patients had undergone the V-shape surgery, and 14 of them with CT images available are included in this study. For comparison, Dr Elefteriades performed a total of 630 traditional ascending aortic procedures during the same time period.

**Table 1:** Demographics of the patients involved in this study

Demographics		Value
Total patient number		14
Age/years, mean ± SD (range)		62.57 ± 12.43 (33–78)
Male, *n* (%)		10 (71.43%)
Max ascending aortic size/cm, mean ± SD (range)		4.41 ± 0.45 (3.50–5.45)
Max aortic root size/cm, mean ± SD (range)		4.53 ± 0.34 (4.00–5.05)
Preoperative aortic insufficiency (AI), *n* (%)		11 (78.57%): 4 mild, 5 moderate, 2 severe
Concomitant procedures	Aortic valve replacement, *n* (%)	11 (78.57%)
	Hemi-arch replacement, *n* (%)	5 (35.71%)
	Total arch replacement, *n* (%)	3 (21.43%)
	Elephant Trunk Stage I, *n* (%)	2 (14.29%)
Hospital mortality, *n* (%)		0 (0%)

AI: aortic insufficiency; SD: standard deviation.

For the 6 patients (P1, P8 and P11–P14) with second follow-up CT used to assess later growth after surgery, the CT of the first time point (Post 1) was obtained within 4 months [median, 2 months (range, 1–4), Table 2] after the surgery. The time interval to the second time point (Post 2) from Post 1 ranged from 20 to 52 months (median, 23.5 months, Table 2). The median follow-up from the surgery date to the Post 2 is 26 months (range, 24–53 months, Table 2) and median follow-up index is 0.79 (range, 0.44–1.0, Table 2). The resolution of the CT images is about 1.2 mm by 1.2 mm by 1.0 mm.

**Table 2:** Effective diameter of the aortic root of Post 1 and Post 2 for 6 patients

	P1	P8	P11	P12	P13	P14
Post 1 (cm)	2.86	3.23	3.63	3.47	3.19	3.52
Post 2 (cm)	2.99	3.42	3.79	3.62	3.33	3.75
Chg (cm)	0.13	0.19	0.16	0.15	0.14	0.23
Chg/year (cm)	0.07	0.10	0.04	0.08	0.08	0.05
$g_{t}$ (%)	4.55%	5.88%	4.41%	4.32%	4.39%	6.53%
$g_{a}$ (%)	2.35%	2.90%	1.21%	2.33%	2.61%	1.47%
${\Delta T}_{01}$ (month)	3	2	2	2	4	1
${\Delta T}_{12}$ (month)	23	24	43	22	20	52
${\Delta T}_{02}$ (month)	26	26	45	24	24	53
${\Delta T}_{0c}$ (month)	26	32	55	31	54	83
FUI	1.0	0.81	0.82	0.77	0.44	0.64

Chg: diameter growth from Post 1 to Post 2; Chg/year: annual diameter growth during Post 1 and Post 2. $g_{t}$: total percentage growth of root effective diameter from Post 1 to Post 2; $g_{a}$: annual percentage growth of root effective diameter during Post 1 and Post 2; ${\Delta T}_{01}:\;$time interval between the surgery date and Post 1; ${\Delta T}_{12}$: time interval between Post 1 and Post 2; ${\Delta T}_{02}:\;$time interval between the surgery date and Post 2; ${\Delta T}_{0c}:\;$time interval between the surgery date and study closure date; FUI: follow-up index.

### Wall stress of the aorta pre- and post-surgery

**Figure 4:**
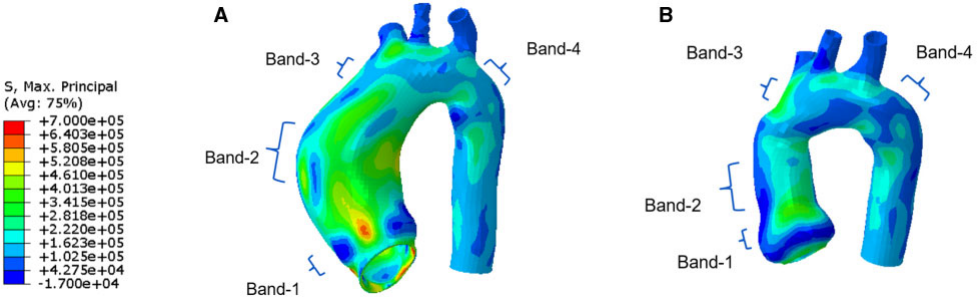
Max-principal stress field of the aorta of a representative patient (P1): (**A**) pre- and (**B**) post-surgery. Four ring bands: band-1 at the repaired aortic root, band-2 at the ascending aorta with the maximum diameter presurgery, band-3 at the ascending aorta just proximal to the brachiocephalic artery (above the graft) and band-4 at the position just distal to the aortic arch.

The wall stress of the aorta of the 10 patients (P1–P10) pre- and post-surgery was obtained from the FE simulation. Figure 4 shows the MP stress field of the aorta of a representative patient (P1) pre- and post-surgery. We extracted the MP stress field of 4 ring bands of the aorta from each patient, with band-1 at the repaired aortic root (from around the sinus to STJ), band-2 at the ascending aneurysm with the maximum diameter presurgery, band-3 at the ascending aorta just proximal to the brachiocephalic artery (essentially, above the graft), which might be slightly affected by the surgery and band-4 at the position just distal to the aortic arch. The presurgery ascending aneurysm of band-2 was surgically replaced by an ascending aortic graft as shown post-surgery. Band-1, band-3 and band-4 post-surgery represent native aortic tissue. The longitudinal height of each ring band presurgery is approximately the same as that post-surgery.

**Figure 5:**
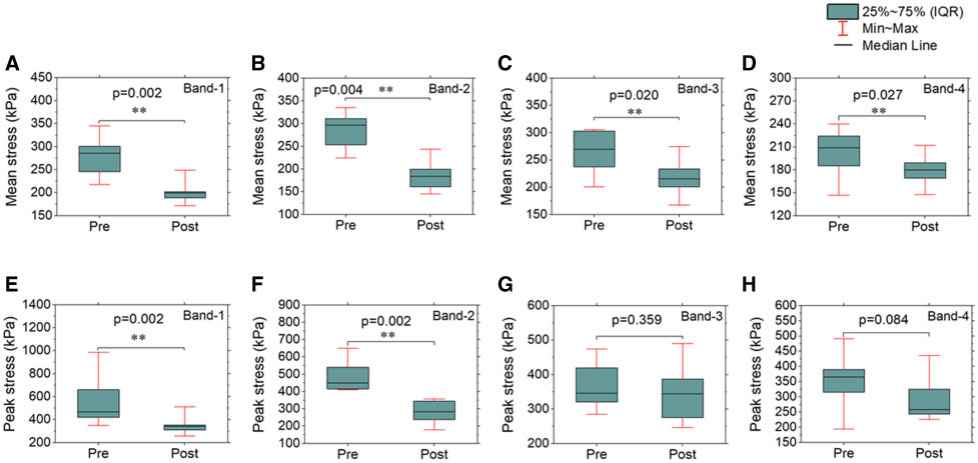
Mean (first row, **A–D**) and peak (second row, **E** and **F**) max-principal stresses of the band-1 (**A**, **E**), band-2 (**B**, **F**), band-3 (**C**, **G**) and band-4 (**D**, **H**). Bar charts represent the median, range and interquartile range (IQR) for the 10 patients. **P-value < 0.05.

Figure 5 shows the mean (first row) and peak (second row) MP stresses of the 4 ring bands between pre- and post-surgery for the 10 patients. There is a significant reduction for the mean and peak MP stresses of band-1, with *P* = 0.002 (Fig. 5A and B). The mean and peak MP stresses of band-2 (Fig. 5A and E) were also reduced significantly, with *P* = 0.004 (Fig. 5B) and *P* = 0.002 (Fig. 5F), respectively. The mean MP stress of band-3 and band-4 has a significant reduction from pre- to post-surgery, with P=0.020 (Fig. 5C) and P=0.027 (Fig. 5D), respectively, while there is no statistical difference between the pre- and post-surgery for the peak MP stress of band-3 (Fig. 5G, P=0.359) and band-4 (Fig. 5H, P=0.027).

### Dilatation of the aortic root post-surgery

**Figure 6:**
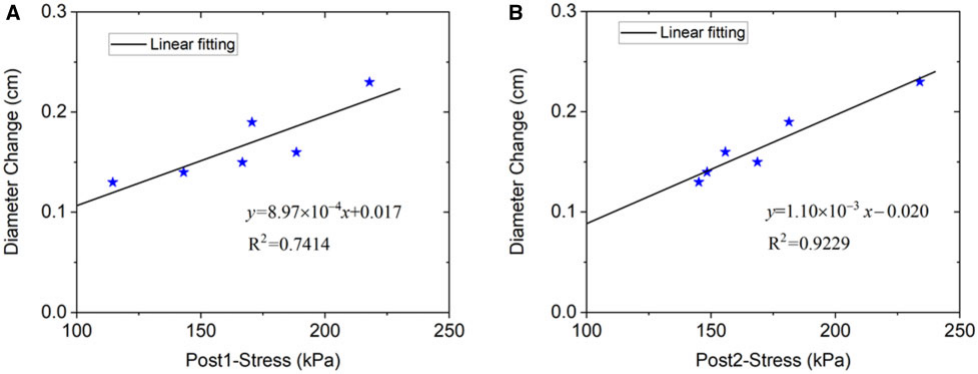
Relation between the diameter change of the aortic root from Post 1 to Post 2 and the mean max-principal stress of the aortic root at Post 1 (**A**) and Post 2 (**B**), for the 6 patients (P1, P8 and P11–P14) shown in Table 2. The blue stars (P1, P8, P12 and P13) have about 2 years interval (range, 20–24 months) between Post 1 and Post 2, and the red stars (P11 and P14) have about 4 years interval (range, 43–52 months) between Post 1 and Post 2.

The effective diameter of the aortic root at Post 1 and Post 2 for the 6 patients (P1, P8 and P11–P14) is presented in Table 2, which showed a minimum annual growth of 1.21% for P11, a maximum annual growth of 2.90% for P8, and an average growth of 5.01% in total, and 2.15%/year. Linear regression trend lines were obtained, with $R^{2}=0.7414$ and 0.9229, for the relations between the diameter change and the mean MP stresses of the root at Post 1 and Post 2, respectively, shown in Fig. 6.

Supplementary Material, Fig. S1 shows the comparison of the effective diameter of the aortic root at 3 time points of presurgery (Pre), Post 1 and Post 2, for 4 patients (P1, P8, P11 and P14) whose the CT images of the aortic root are available for all the 3 time points. The effective diameter of the aortic root of Post 2 within 4 years after the surgery is smaller than that of presurgery for all four patients.

## DISCUSSION

We obtained the static stress distribution of the aortic wall under patient-specific systolic pressure. The cyclic loading and unloading were not considered. Based on the principles of biomechanics, larger pressure loading would induce larger stress in the aortic wall. The wall stress under systolic pressure is expected to be maximum; and further, the corresponding risk is also the maximum, during a cardiac cycle. Thus, we only considered the state of systolic pressure.

Detailed stress distribution of the aortic wall from band-1 to band-4 can be compared between pre- and post-surgery states, using our patient-specific stress analysis. The V-shape surgery significantly reduced the mean and peak MP stress of band-1 (corresponding to the repaired aortic root; Fig. 5A and E). If the rupture strength of the root wall is assumed to be unchanged post-surgery, the rupture risk will be expected to decrease in concert with the decreased wall stress [10–12]. The major portion of the ascending aneurysm (band-2 in Fig. 4A) was replaced by an ascending aortic graft (band-2 in Fig. 4B). The diameter of the graft is much smaller than that of the ascending aneurysm presurgery and the stress of band-2 was significantly reduced by the surgery (Fig. 5B and F). Although the polymeric graft has no rupture issue, the reduction of the diameter of band-2 may contribute to the stress reduction (Fig. 5C) of the adjacent band-3 which is native aortic tissue. The average value for the 10 patients of the mean and peak MP stress of band-4 (Fig. 5D and H) also decreases after the surgery, but there is no statistical difference (P ≥ 0.05) between the pre- and post-surgery for the stress of band-4.

It has been shown by Elefteriades *et al.* [5] that the mean wall tension of the aortic root decreased post-surgery with the reduced root diameter, simply based on the Laplace’s law. However, the change of the wall stress, especially the peak wall stress, of the aortic root may not have a similar simple relation with the reduced diameter, since the wall stress field is usually inhomogeneous and depends not only on the diameter but also on other shape features including the centreline and surface curvatures, as has been shown by Liang *et al.* [23] and de Galarreta *et al.* [28]. Thus, a detailed patient-specific biomechanical analysis in this study is essential for a more accurate evaluation for the impact of the novel V-shape surgery on the wall stress of the aortic root.

Shang *et al.* [17] investigated the relation between the wall stress and expansion rate in descending thoracic aortic aneurysms without surgery, and they found that larger peak wall stress results in larger aneurysm expansion rate. However, there is currently a lack of studies on the dilatation of aortic wall/root after a surgical operation. Thus, it is also essential to quantitatively estimate the potential aortic root dilatation after the surgery for a comprehensive evaluation of the novel V-shape surgery.

The results of this study indicated that the aortic root manifests only small dilatation post-surgery. The average percentage diameter increase is 5.01% in total, and 2.15%/year, and the average absolute increase of 1.7 mm in total and 0.71 mm/year, which is much smaller than the diameter increase reported in the literature for aortic aneurysms without surgery (e.g. a mean diameter increase of 2.9 mm/year obtained in Shang *et al.* [17], and 4.2 mm/year in Hirose *et al.* [29]). The dilatation could be affected by the wall stress since larger root wall stress results in larger root dilatation (as shown in Fig. 6). Moreover, the comparison of results in Fig. 6A and B indicates that the impact of wall stress is relatively constant over time.

In addition, it should be noted that, although the effective diameter of the aortic root of Post 2 increases mildly comparing with that of Post 1, the root diameter of Post 2 is still smaller than that of presurgery at current stage (within 4 years after the surgery), which suggests the V-shape surgery effectively reduces the aortic root diameter for a relatively long time period post-surgery.

This advanced engineering analysis adds credence to the V-shaped resection of the non-coronary sinus as a viable procedure. We tend to use the V-shaped resection for aortic roots in the range of 4.0–5.0 cm in patients who are not candidates for full aortic root replacement because of age or frailty or need for extensive, critical other intraoperative procedures (e.g. mitral valve surgery, coronary artery bypass, aortic arch replacement, etc.). We do not use it for aortic roots beyond 5.0 cm. The beneficial findings in this engineering analysis give support for the V-shaped resection as another tool in the surgeon’s armamentarium for the mildly dilated aortic root.

One limitation of this study is that the analyses are based on a relatively small cohort of patients (total *n* = 14), since the V-shape surgery was the exception rather than the rule and was reserved largely for elderly or infirm patients. However, results were quite consistent among this small group of patients, suggesting that these results would generalize to larger cohorts. For example, the mean and peak values of the root wall stress (band-1) for all 10 patients was reduced by more than −14% after the surgery, which results in very small *P*-values (Fig. 5A and E) between pre- and post-surgery. Thus, it can be expected that similar results may be obtained with a larger cohort of patients. Finally, a uniform thickness of 1.5 mm for the aortic wall was applied in the FE simulation, while the thickness of the aortic wall may be non-uniform. The wall thickness may also influence the wall stress. However, the wall thickness for a specific patient may not change much from pre- to post-surgery, and thus the trend of the stress change from pre- to post-surgery should differ not much when patient-specific wall thickness is applied. In the future, *in vivo* non-uniform thickness of the aortic wall may be obtained based on the method in Elefteriades *et al.* [30] when both contrast and non-contrast CT scans are available.

## CONCLUSIONS

This study marks the first biomechanical analysis of the novel V-shape surgery. The study has demonstrated a significant reduction in aortic wall stress of the aortic root repaired by V-shaped resection of the moderately dilated aortic root at the time of ascending aortic replacement. Wall stress reduction is important in reducing the dilatation process. Indeed, the low redilatation rate (2.15%/year) portends well for the future of the aortic root repaired by V-shaped resection.

## SUPPLEMENTARY MATERIAL

Supplementary material is available at *ICVTS* online.

## Supplementary Material

ivac004_Supplementary_Material

## ABBREVIATIONS

3D: Three-dimensional
CT: Computed tomography
FE: Finite element
MP: Max-principal
STJ: Sinotubular junction
