# Supplementary material for: Engineering analysis of aortic wall stress and root dilatation in the V-shape surgery for treatment of ascending aortic aneurysms
Source: Interact Cardiovasc Thorac Surg. 2022 Feb 3;34(6):1124–31. doi: 10.1093/icvts/ivac004 (PMC9159430; doi:10.1093/icvts/ivac004)

**Supplementary Materials for:**

**Engineering Analysis of Aortic Wall Stress and Root Dilatation in the V-shape Surgery for Treatment of Ascending Aortic Aneurysms**

Hai Dong^1^, Minliang Liu^1^, Tongran Qin^1^, Liang Liang^2^, Bulat Ziganshin^3^, Hesham Ellauzi^3^, Mohammad Zafar^3^, Sophie Jang^3^, John Elefteriades^3^, Wei Sun^1^

^1^ Tissue Mechanics Laboratory, The Wallace H. Coulter Department of Biomedical Engineering

Georgia Institute of Technology and Emory University, Atlanta, GA;

*^2^Department of Computer Science, University of Miami, Coral Gables, FL;*

^3^Aortic Institute at Yale-New Haven Hospital,

Yale University School of Medicine, New Haven, CT

S1. Finite Element Analysis Based on Static Determinacy Approach

In this study, we performed finite element (FE) simulation of the aorta by the Abaqus/Standard 2019 (SIMULIA, Providence, RI). Since the aortic wall stress is independent of material properties, during the FE simulation, the simple isotropic linear elastic material properties of extremely large stiffness were applied to the aortic wall, with the two fundamental material parameters Young’s modulus to be $20 \mathrm{GPa}$ and the Poisson’s ratio to be $0.49$. The circumferential and axial displacements were fixed at the boundaries, including the inlet of the ascending aorta (around the aortic annulus), outlet of the three branches, and outlet of the descending aorta. The radial displacement at the boundaries was set to be un-constrained. The patient-specific systolic blood pressure at (or near) the date of the CT scan was applied to the inner surface of the aorta during the FE simulation. The systolic stress fields of the aorta pre- and post-surgery were extracted and analyzed.

S2. Calculation of the effective diameter and growth rate of the aortic root

The effective diameter ($D_{e}$) of the root was determined by $V=L (\pi D_{e}^{2}/4)$ where $V$ is the root enclosed volume and $L$ is the root longitudinal height. Further, we have

$$D_{e}=2\sqrt{\frac{V}{\pi L}}, (S1)$$

which suggests that the effective diameter is proportional to the square root of the volume divided by the height. Once we obtained the effect diameter $\boldsymbol{D}_{\boldsymbol{e1}}$ of the root at Post1 and $\boldsymbol{D}_{\boldsymbol{e2}}$ at Post2, the annual percentage growth $\boldsymbol{g}_{\boldsymbol{a}}$ and total percentage growth $\boldsymbol{g}_{\boldsymbol{t}}$ for each patient were calculated by solving the equations $\boldsymbol{D}_{\boldsymbol{e1}}{\boldsymbol{(1+}\boldsymbol{g}_{\boldsymbol{a}}\boldsymbol{)}}^{\boldsymbol{n}}\boldsymbol{=}\boldsymbol{D}_{\boldsymbol{e2}}$ and $\boldsymbol{D}_{\boldsymbol{e1}}\boldsymbol{(1+}\boldsymbol{g}_{\boldsymbol{t}}\boldsymbol{)=}\boldsymbol{D}_{\boldsymbol{e2}}$, where $\boldsymbol{n}$ is the time interval between Post1 and Post2 in years. The average annual and total percentage growth were obtained by averaging the annual and total growth of the 6 patients in the follow-up study.

**Fig S1.** Comparison of the effective diameter of the aortic root pre-surgery (Pre) and post-surgery (Post1 and Post2), for P1, P8, P11 and P14. The effective diameter of the aortic root of Post2 within 4 years after the surgery is smaller than that of pre-surgery for all four patients.


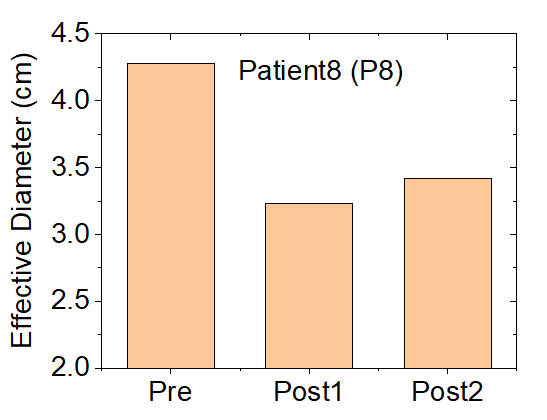

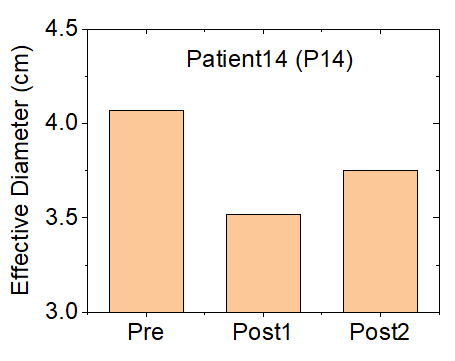

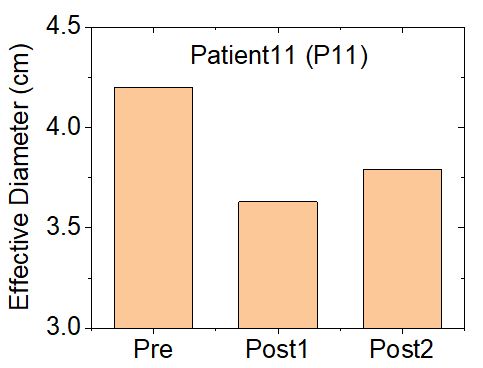

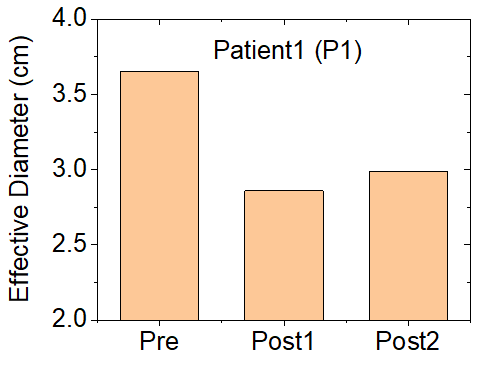

Supplement: ivac004_Supplementary_Material [file ivac004_supplementary_material.docx]
